# Identification of microbial metabolites that accelerate the ubiquitin-dependent degradation of c-Myc

**DOI:** 10.32604/or.2023.030248

**Published:** 2023-07-21

**Authors:** ZIYU LIU, AKIKO OKANO, EMIKO SANADA, YUSHI FUTAMURA, TOSHIHIKO NOGAWA, KOSUKE ISHIKAWA, KENTARO SEMBA, JIANG LI, XIAOMENG LI, HIROYUKI OSADA, NOBUMOTO WATANABE

**Affiliations:** 1 Bioprobe Application Research Unit, RIKEN CSRS, Saitama, 351-0198, Japan; 2 Graduate School of Medical and Dental Sciences, Tokyo Medical and Dental University, Tokyo, 113-8510, Japan; 3 Chemical Biology Research Group, RIKEN CSRS, Saitama, 351-0198, Japan; 4 Chemical Resource Development Research Unit, RIKEN CSRS, Saitama, 351-0198, Japan; 5 Molecular Structure Characterization Unit, RIKEN CSRS, Saitama, 351-0198, Japan; 6 Japan Biological Informatics Consortium (JBIC), Tokyo, 135-8073, Japan; 7 Department of Life Science and Medical Bioscience, School of Advanced Science and Engineering, Waseda University, Tokyo, 162-8480, Japan; 8 Medical-Industrial Translational Research Center, Fukushima Medical University, Fukushima, 960-1295, Japan; 9 Guangdong Engineering Research Center of Oral Restoration and Reconstruction, Affiliated Stomatology Hospital of Guangzhou Medical University, Guangzhou, China; 10 KingMed School of Laboratory Medicine, Guangzhou Medical University, Guangzhou, China; 11 Department of Pharmaceutical Sciences, University of Shizuoka, Shizuoka, 422-8526, Japan

**Keywords:** High-throughput screening, Phosphorylation, Reactive oxygen species

## Abstract

Myc belongs to a family of proto-oncogenes that encode transcription factors. The overexpression of c-Myc causes many types of cancers. Recently, we established a system for screening c-Myc inhibitors and identified antimycin A by screening the RIKEN NPDepo chemical library. The specific mechanism of promoting tumor cell metastasis by high c-Myc expression remains to be explained. In this study, we screened approximately 5,600 microbial extracts using this system and identified a broth prepared from *Streptomyces* sp. RK19-A0402 strongly inhibits c-Myc transcriptional activity. After purification of the hit broth, we identified compounds closely related to the aglycone of cytovaricin and had a structure similar to that of oligomycin A. Similar to oligomycin A, the hit compounds inhibited mitochondrial complex V. The mitochondria dysfunction caused by the compounds induced the production of reactive oxygen species (ROS), and the ROS activated GSK3α/β that phosphorylated c-Myc for ubiquitination. This study provides a successful screening strategy for identifying natural products as potential c-Myc inhibitors as potential anticancer agents.

## Introduction

Myc belongs to a family of proto-oncogenes that encode transcription factors. c-Myc and Max form a heterodimer and carry out the transcription of genes important for regulating cell growth. c-Myc is a critical regulator of cell proliferation and growth. c-Myc regulates 10%–15% of genes in higher organisms, from *Drosophila* to humans, by recruiting histone acetylases, chromatin regulatory proteins, basic transcriptional regulators, and DNA methylases [1–4]. c-Myc participates in various cellular processes, including cell cycle control, metabolism, protein synthesis, cell adhesion, cytoskeleton scaffolding, apoptosis, and vascular formation [1,5]. c-Myc deregulation is a signature of over 70% of human cancers [4,6–8].

Therapeutic strategies are developed for compounds that inhibit c-Myc protein directly or indirectly [9]. The mechanisms to directly target c-Myc usually block the interaction between c-Myc and MAX (MYCi361, JQ1, I-BET, and OTX015) [10–13]. The indirect targeting c-Myc treatment involves regulating the signaling pathway or mechanism to weaken the expression of c-Myc, such as epigenetic silencing of its gene, transcriptional, post-transcriptional, and so on [14–16]. Due to the lack of follow-up clinical studies and drug resistance, the therapeutic effect of c-Myc inhibitors has not been widely used. The active search for compounds for c-Myc-driven tumor therapy remains urgent. Notably, many indirect inhibitors of c-Myc have been tested in clinical trials; however, problems such as drug resistance still need to be solved [17].

The ubiquitin-proteasome pathway is the major pathway through which c-Myc levels are controlled [18,19]. The synergy mediates ubiquitination between the following three enzymes: ubiquitin-activating enzyme (E1), ubiquitin transferase (E2), and ubiquitin ligase (E3) [20,21]. Fbw7 is one of the recognition subunits for c-Myc E3 ubiquitin ligase and preferentially interacts with c-Myc, which is double-phosphorylated at threonine 58 (T58) and serine 62 (S62) [22–24]. The Ras-ERK signaling pathway regulates the phosphorylation of S62 and is necessary for the phosphorylation of T58 [25]. The phosphorylation of T58 is regulated by glycogen synthetic kinase 3 (GSK3) [26]. Phosphorylation of S62 alone stabilizes c-Myc. The subsequent phosphorylation of T58 promotes c-Myc [26]. Considering the importance of T58 phosphorylation, this site’s regulation mechanism is also significant for c-Myc protein stability.

High c-Myc expression causes tumorigenesis by deregulating genetic and epigenetic checkpoint mechanisms and controlling cell proliferation, apoptosis, and differentiation. Recent studies have also revealed that high c-Myc expression in tumor cells promotes tumor cell metastasis, although the specific mechanism remains unexplained [27]. Similarly, many studies have shown that abnormally expressed c-Myc promotes tumor cell proliferation and stimulates tumor cell tolerance to hypoxic environments [28]. In chemotherapy-resistant cancers, c-Myc increases mitochondrial oxidative phosphorylation (OXPHOS) and ROS [19]. Moreover, the overexpression of c-Myc reduces the sensitivity of cells to chemotherapy and causes great resistance to prognostic treatment in patients with tumors.

Recently, we found that the complex III inhibitor antimycin A degrades c-Myc protein and increases ROS levels to inhibit c-Myc-enriched cancer cell growth (manuscript under revision). The inhibition of cell growth by antimycin A is caused by ROS-dependent and ROS-independent pathways. Only the ROS-dependent pathway was activated in the presence of c-Myc, which appears to be specific to cancer cells. In this study, we screened more than 5,600 microbial extracts using a cell-based high-throughput screening system. We isolated the active compounds from a hit broth extract, and the planar structure was identified as SS49, which showed OXPHOS inhibitory activity as an oligomycin. The hit compounds enhanced the degradation of the c-Myc protein by activating GSK3 by ROS from the damaged mitochondria. This study provides a successful screening strategy for identifying natural products as potential c-Myc inhibitors as potential anticancer agents.

## Materials and Methods

### Cell lines

Reporter cell lines that respond to the expression and transcriptional activity of c-Myc (E-H1) or HNF1B (D-D1) derived from NMuMG cells were cultured in Dulbecco’s modified Eagle’s medium (DMEM; Gibco, Grand Island, NY, USA) with 10% fetal bovine serum (FBS; Sigma-Aldrich, St. Louis, MO, USA), supplemented with 1% insulin (FUJI FILM Wako Pure Chemical, Osaka, Japan) and 0.5% penicillin-streptomycin (P/S; Invitrogen, Carlsbad, CA, USA) [29]. The E-H1 and D-D1 cells express c-Myc and HNF1B under the Tet-ON system and the fluorescent protein monomeric Keima (mKeima). Since the ORF of mKeima was inserted after the c-Myc ORF or HNF1B following the internal ribosomal entry site, c-Myc or HNF1B expression could be monitored through the mKeima expression. These proteins were induced by the addition of Doxycycline (DOX, 100 ng/mL) [29]. The cancer cell lines HeLa, PANC-1, MIA PaCa-2, DU145, and A549 were cultured in DMEM supplemented with 10% FBS and 0.5% P/S, and HCT116 and HL-60 cells were grown in Roswell Park Memorial Institute (RPMI) 1640 medium (Gibco) supplemented with 10% FBS and 0.5% P/S. Cells were incubated at 37°C in a humidified atmosphere containing 5% CO_2_.

### Broth culture and extracts

Actinomycete and fungal strains were isolated from soil samples. Thousands of actinomycete and fungal strains were cultured in different media, and their culture broths were prepared and used for screening.

### Strain and culture

The actinomycete, *Streptomyces* sp. RK19-A0402, was isolated from a soil sample collected in Hachioji, Tokyo, Japan, in 2018 and identified using 16S rDNA sequencing (Techno Suruga Laboratory, Shizuoka, Japan). The strain was precultured in a 500 mL cylindrical flask containing 70 mL of SY medium (0.1% yeast extract, 1% soluble starch, and 0.1% NZ-amine type A) at 28°C for 2 days on a rotary shaker at 150 rpm. Then, 4 mL of the preculture was incubated in five 2 L Erlenmeyer flasks containing 300 mL of TYB medium (0.5% tryptone and 0.3% yeast extract) at 28°C for 5 days on the same rotary shaker at 150 rpm.

### Extraction and isolation

Large-scale culture broth (1.5 L) was extracted with the same volume of acetone and filtered to remove the mycelia. The acetone extract was evaporated under reduced pressure to obtain a water suspension. It was then partitioned thrice using the same volume of EtOAc. The EtOAc extract was evaporated under reduced pressure to obtain 180 mg of brown gum. The gum was subjected to SiO_2_-medium pressure liquid chromatography (MPLC) with stepwise elution using CHCl_3_/MeOH (CHCl_3_/MeOH = 100:0, 99:1, 98:2, 95:5, 90:10, 80:20, 50:50, and 0:100) to obtain eight fractions. The fourth fraction, eluted at a 95:5 ratio, was subjected to C18-MPLC with a MeOH/H_2_O linear gradient system to obtain four subfractions. The third subfraction was separated by C18-MPLC using a MeCN/H_2_O linear gradient system to obtain a crude mixture of compounds **1** and **2**. It was separated and purified by C18-HPLC with an isocratic elution of MeCN/H_2_O (1:1) to afford 1.4 and 3.3 mg of compounds **1** and **2**, respectively. After C18-HPLC purification, compounds **1** and **2** showed identical UV chromatograms by liquid chromatography-mass spectrometry (LC-MS) analysis at approximately 1:4.

**Compound 1:** UV (MeCN/H_2_O diode array detector (DAD)-LC-MS analysis) λ_max_ 215sh nm; high-resolution electrospray ionization time-of-flight mass spectrometry (HR–ESI–TOFMS) found *m/z* 747.4643 [M+Na]^+^ calculated for 747.4654 C_40_H_68_O_11_Na; ^13^C nuclear magnetic resonance (NMR) chemical shifts are summarized in Suppl. Table S1.

**Compound 2:** UV (MeCN/H_2_O DAD–LC–MS analysis) λ_max_ 215sh nm; HR–ESI–TOFMS found *m/z* 747.4645 [M+Na]^+^ calculated for 747.4654 C_40_H_68_O_11_Na; ^1^H and ^13^C NMR chemical shifts are summarized in Suppl. Table S1.

**Mixture of 1 and 2:** [α]_D_^26^ −30° (*c* 0.1, MeOH); UV (MeOH) λ_max_ (log ε) 212sh (4.21) nm; IR ν_max_ (ATR) 3353, 2929, 1698, 1616, 1457, 1351, 1270, 1062, 1016, 966 cm^–1^.

### General experimental methods

All solvents and reagents were of analytical grade and purchased from commercial sources. The UV spectra and optical rotations were recorded using a UV-1900i UV-Vis spectrophotometer (Shimadzu Corporation, Kyoto, Japan) and a HORIBA SEPA-300 high-sensitive polarimeter (HORIBA, Kyoto, Japan). The IR spectra were recorded using an FT-720 spectrometer equipped with a DuraSampl IR II ATR instrument (HORIBA). MPLC was performed using the Teledyne ISCO CombiFlash Companion (Teledyne ISCO, Lincoln, NE, USA). Preparative HPLC was conducted using a Waters 600 pump system (Waters, Milford, MA, USA) equipped with a Cosmosil MS-II C18 column (5 μm, 10 mm i.d. × 250 mm; Nacalai, Kyoto, Japan). DAD-LC-MS analyses were conducted using a Waters UPLC H-class system with a Waters UPLC BEH C18-column (1.7 μm, 2.1 mm i.d. × 50 mm; Waters) connected to an AB Sciex API3200 MS/MS system (AB Sciex, Framingham, MA, USA) equipped with an electrospray ionization (ESI) probe. HR–ESI–TOFMS was measured using a Vion IMS QTOF Mass Spectrometer (Waters). NMR spectra were recorded using a JEOL ECA500 FT-NMR spectrometer (JEOL, Tokyo, Japan) at 500 MHz for ^1^H NMR and 125 MHz for ^13^C NMR. The chemical shifts were referenced to the corresponding solvent signals (δ_H_ 7.24 and δ_C_ 77.23 in CDCl_3_).

### Screening procedure

E-H1 cells (1 × 10^4^ cells) were seeded in 96-well plates 1 d before sample addition. Then, 1 μL of the sample (conc. = 1/10 of original broth samples) together with 2.5 μL of doxycycline (DOX; 8 μg/mL) were added to 200 μL of medium and incubated for 24 h. After that, the medium was discarded, and cells were fixed with 200 μL of 3.7% formaldehyde in phosphate-buffered saline (PBS) for 15 min, after which the cells were washed with PBS and stained with 100 μL of Hoechst 33342 (1 μg/mL in PBS; Sigma-Aldrich). Subsequently, the fluorescence levels of enhanced green fluorescent protein, mKeima, and Hoechst were measured using the IN Cell Analyzer 2000 (INCA; GE Healthcare, Chicago, IL, USA) at the setting of FITC, Texas red, and DAPI, respectively, and analyzed. Images were quantified using IN Cell Developer Toolbox 1.9.2 (GE Healthcare).

### Western blotting analysis

E-H1 and cancer cells were seeded at 3 × 10^5^ cells/well in a 6-well plate, cultured with 3 mL of appropriate medium containing 10% FBS per well, and incubated in a 37°C incubator. After 24 h, the medium was changed, and 1 μL samples were added to 3 mL of medium (with or without 100 ng/mL DOX for E-H1 cells) for an appropriate time. To inhibit GSK3, specific inhibitor CT99021 (Cayman Chemical Company, Ann Arbor, MI, USA, #13122) was used.

Next, treated cells were washed with 1 mL of pre-cooled PBS at 4°C twice, after which 500 μL of PBS was added to each well, and cells were scraped into a 1.5 mL centrifuge tube and harvested by centrifugation at 5,000 rpm for 3 min. Subsequently, the cells were lysed in RIPA buffer and centrifuged at 15,000 rpm for 10 min to collect the supernatant protein lysate. Finally, the protein concentration was quantified using the Pierce BCA Protein Assay kit (Thermo Fisher Scientific, Waltham, MA, USA).

Cell lysates were separated by sodium dodecyl-sulfate polyacrylamide gel electrophoresis (5% stacking gel and 7.5% separating gel) and transferred onto polyvinylidene fluoride membranes (Millipore, Billerica, MA, USA). Next, the membrane was blocked with 5% milk in Tris-buffered saline with 0.1% Tween 20 for 1 h at 4°C and incubated overnight at 4°C with anti-c-Myc (1:1,000, #ab32072; Abcam, Cambridge, UK), anti-HNF1B (1:2000, Home made, manuscript in preparation), anti-GSK3α/β (1:1,000, #5676; Cell Signaling Technology, Danvers, MA, USA), anti-GSK3α/β-phosphor-S21/9 (1:1000, #8566; Cell Signaling Technology), or anti-GSK3β-phosphor Thr390 (1:1000, #3548; Cell Signaling). The membrane was washed and incubated with the appropriate secondary HRP-conjugated antibodies for 1 h at 25°C. After washing, the bound antibodies were detected using the SuperSignal West Pico chemiluminescent substrate (Thermo Fisher Scientific) and FUSION SOLO S (Vilber, Marne-la-Vallée, France). Western blot results were quantified using ImageJ software (NIH, Bethesda, MD, USA). Membranes were stained with CBB to confirm equal loading.

### Analyses of ubiquitination of c-Myc

E-H1 and HCT116 cells were treated with samples, DOX (100 ng/mL), and MG132 (6 μM) for 6 h and lysed in lysis buffer (20 mM Tris-HCl [pH 8.0], 100 mM NaCl, 1 mM EDTA, and 0.5% Nonidet P-40). Next, the cell lysate was incubated with anti-c-Myc-antibody (9B11)-conjugated beads (#3400; Cell Signaling Technology) overnight at 4°C. After that, the beads were washed thrice with lysis buffer, and the bound proteins were eluted and analyzed by western blotting using an anti-ubiquitin antibody (#3933S; Cell Signaling Technology).

### Mitochondrial activity assay

HCT116 cells (1 × 10^4^ cells/well) were seeded in 96-well plates and incubated overnight before sample addition. Then, 1 μL of samples were added and incubated for 2 h in a 37°C CO_2_ incubator. Then the medium was changed to RPMI/NaHCO_3_ free (containing samples) and incubated for 1 h in a 37°C non-CO_2_ incubator, after which the plate was loaded in the XFe analyzer (Agilent, Santa Clara, CA, USA). After measurement of the oxygen consumption rate (OCR) and extracellular acidification rate (ECAR) in the presence of test compounds, the Cell Mito Stress Test (Agilent) was performed, in which OCR values were measured after the addition of oligomycin A (1 μM), carbonyl cyanide 4-(trifluoromethoxy)-phenylhydrazone (FCCP; 0.125 μM), and rotenone/antimycin A (1 μM each) in a stepwise manner.

### Mitochondrial ROS detection assay

According to the manufacturer’s instructions, mitochondrial ROS levels were measured using a Mitochondrial ROS Detection Assay Kit (Cayman Chemical Company, Ann Arbor, MI, USA). HCT116 cells (1 × 10^4^ cells/well) were seeded in 96-well plates 1 d before sample addition. After discarding the medium, 120 μL of cell-based assay buffer was added to each well. Furthermore, the buffer was changed to 100 μL of test ROS reagent staining and incubated at 37°C for 20 min, after which the cells were washed with Hanks’ balanced salt solution (HBSS) thrice, and 10 μL of the compound solution was added to 200 µL of HBSS. Following incubation at 37°C for 1 h, the cells were fixed (3.7% formaldehyde, 200 μL/well) for 15 min, washed with PBS, and stained with Hoechst 33342 (100 μL of 1 ug/mL in PBS). The fluorescence of the ROS reagent and Hoechst stain was measured using the IN Cell Analyzer 2000 (INCA; GE Healthcare) with FITC (Ex)/Cy3 (Em) and DAPI sets, respectively. Idebenone (Tokyo Chemical Industry Co., Ltd., Tokyo Japan #I0848) was used to reduce the ROS level.

### Cell growth assay

HCT116 cells (1 × 10^4^ cells) were seeded in 96-well plates 1 day before sample addition. Test samples were added to 200 μL of medium and incubated for 48 h. Subsequently, the medium was discarded, cells were fixed, and the number of nuclei stained with Hoechst dye was measured using the INCA with the DAPI filter set.

### Statistical analysis

Data presented as means ± standard deviation of the mean (SDM). The statistical analyses were performed using the “ANOVA test” in R (R Foundation for Statistical Computing, Vienna, Austria) (*p < 0.05, **p < 0.01, ***p < 0.001). All the results were selected from at least two independent experiments.

## Results

### Development of a screening system to identify c-Myc transcription activity inhibitors

The high-throughput screening system was developed to efficiently screen c-Myc inhibitors using E-H1 cells [29], in which the fluorescence of mKeima and EGFP can monitor the expression and transcription activity of c-Myc. The details of the system will be published elsewhere (manuscript under revision). Another negative control cell with an unrelated transcription factor (HNF1B) instead of c-Myc was used to confirm the specific inhibitory effect of compounds.

### Screening and identification of c-Myc inhibitor from microbial metabolites

While screening approximately 5,600 microbial extracts using the high-throughput screening system for c-Myc inhibitors, we found that the extracts from the actinomycetes strain *Streptomyces* sp. RK19-A0402 exhibited potent inhibitory activity. An EtOAc-soluble extract from a 1.5 L culture broth of the strain was separated by a combination of MPLC and HPLC to afford compounds **1** and **2**. These compounds also exhibit inhibitory activity without affecting the expression of mKeima, indicating that these compounds did not affect the translation of the c-Myc protein (Suppl. Fig. S1). After purification, compounds **1** and **2** showed identical UV chromatograms at a ratio of approximately 1:4, according to LC-MS analysis.

Compounds **1** and **2** showed identical UV and mass spectra, and the molecular formula of both compounds was determined to be C_40_H_68_O_11_ using HR–ESI–TOFMS, suggesting that they were tautomers or isomers in the solvent. Therefore, the structure was investigated as a mixture, focusing on major compound **2**. The ^1^H NMR spectrum of compound **2** showed seven methyl signals, including five doublets, one triplet, and one singlet; most of each signal overlapped with an identical small signal derived from minor compound **1**. The ^13^C NMR spectrum also showed doubling signals for most carbon atoms, with an intensity of approximately 4:1. These observations supported the hypothesis that **1** and **2** are isomers. The structure was elucidated by focusing on the major signals corresponding to **2**. The ^13^C NMR spectrum included a carbonyl carbon at 165.3 ppm, four olefin signals, a characteristic methine signal at 101.5 ppm, and a quaternary signal at 97.4 ppm, suggesting that **2** possessed two hemiacetal or similar moieties. These spectral features are related to oligomycins. Next, 2D NMR spectra, including heteronuclear single quantum coherence spectroscopy (HSQC), double quantum filtered-correlation spectroscopy (DQF-COSY), and heteronuclear multiple bond correlation (HMBC), were used to construct the structure. HSQC correlations confirmed direct connections between the protons and carbon. The proton spin networks were confirmed by the correlations observed in DQF-COSY.

**Figure 1:**
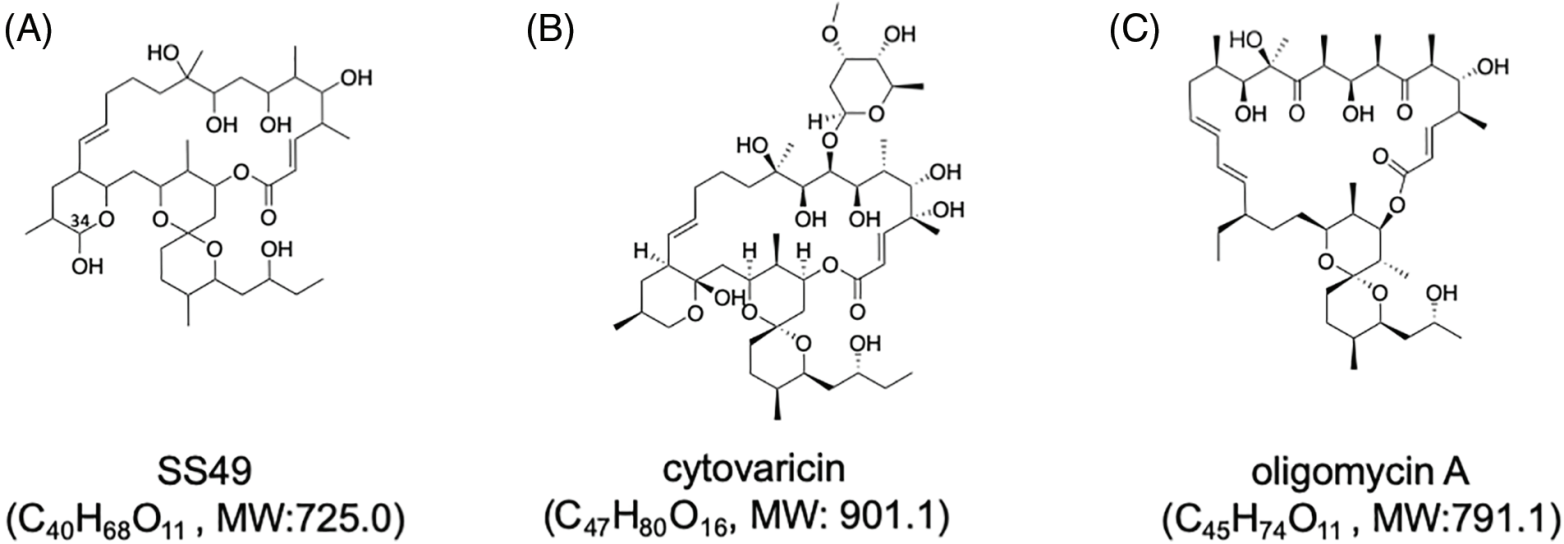
Structures of SS49 (A), cytovaricin (B), and oligomycin A (C). Molecular formulae with molecular weights (MWs) of each compound are shown. The position of C-34 of SS49 is shown in (A).

The entire structure was constructed using HMBC correlations considering ^13^C NMR chemical shift values and the index of hydrogen deficiency. Based on these results, the structure of **2** was determined. The planar structure was found to be identical to that of SS49 [30] (Fig. 1A). SS49 is closely related to cytovaricin aglycone [31]. It has a structure similar to that of oligomycin A [32] (Figs. 1B and 1C). Compound **1** appeared to have the same planar structure as **2** because of its almost identical ^13^C NMR chemical shifts. However, the ^1^H NMR spectrum showed an unidentified small characteristic signal at 4.97 ppm as a broad singlet connected to a small ^13^C methine signal at 94.8 ppm. These chemical shift values suggested that this signal was a hemiacetal carbon and was assigned to the C-34 position (Fig. 1A, Suppl. Table S1). Based on these observations, **1** may be an epimer of **2** at the hydroxyl group at C-34.

**Figure 2:**
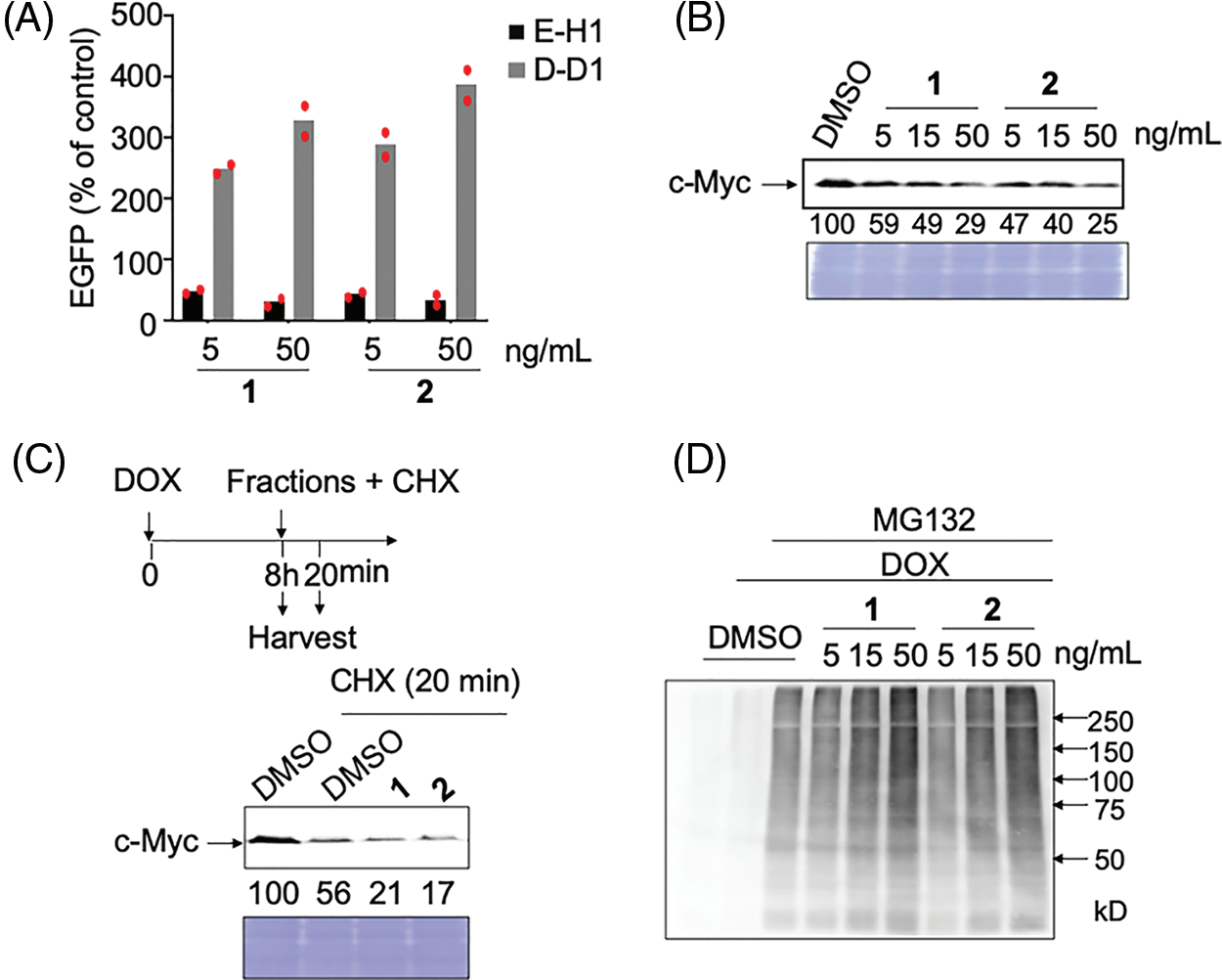
Analyses of the mechanism of action of c-Myc inhibitors, compounds **1** and **2**. (A) c-Myc-specific transcriptional inhibition by hit compounds **1** and **2**. E-H1 and D-D1 cells were fixed for 24 h after adding 100 ng/mL doxycycline (DOX) and **1** or **2**. The relative values (% of control [DOX+, dimethyl sulfoxide (DMSO)]) as mean are shown. Results were obtained from two independent experiments. The red dots indicate individual measurements. (B) Dose-dependent effects of hit compounds **1** and **2** on c-Myc levels in E-H1 cells. E-H1 cells were harvested and lysed after treatment with 100 ng/mL DOX and compounds **1** or **2** for 24 h, and c-Myc levels were analyzed by immunoblotting. Coomassie brilliant blue (CBB) staining was used as the loading control. Protein levels were quantified using ImageJ software and are shown as the percentage of cells treated with DMSO. Results were obtained from two independent experiments. (C) c-Myc degradation by hit compounds **1** and **2** was analyzed using cycloheximide (CHX) chase. E-H1 cells were treated with 50 ng/mL compounds **1** or **2** with 40 μg/mL CHX 8 h after 100 ng/mL DOX addition, then harvested at 0 and 20 min. c-Myc levels were analyzed by immunoblotting. CBB staining was used as the loading control. Protein levels were quantified using ImageJ and are shown as the percentage of cells treated with DMSO at 0 h (8 h). The figure is representative of two independent experiments. (D) The ubiquitination of c-Myc by hit compounds **1** and **2** increased in E-H1 cells dose-dependently. Cells were harvested and lysed after treatment with DOX (100 ng/mL), compounds **1** and **2**, and MG-132 (6 μM) for 6 h. c-Myc proteins in each sample were harvested from 300 μg of lysate using anti-c-Myc antibody-conjugated beads, and their ubiquitination was analyzed by immunoblotting.

The specific activities of the two compounds were similar (Fig. 2A). These compounds had no inhibitory activity on another unrelated transcription factor (HNF1B) in D-D1 cells, indicating that they did not inhibit transcription (Fig. 2A). Rather, these compounds increased the expression of EGFP in D-D1 cells. We confirmed that compounds **1** and **2** do not increase the level of HNF1B in HCT116 cells (Suppl. Fig. S2). Therefore, the mechanism of this increase is still unclear.

We examined whether these compounds inhibited c-Myc activity by degrading c-Myc, as observed after treatment with antimycin A (manuscript under revision). As expected, after treatment with these two compounds, the c-Myc protein levels in E-H1 cells decreased dose-dependent (Fig. 2B). To confirm that this decrease was caused by accelerated protein degradation, we performed a cycloheximide chase assay. Compound treatment decreased the c-Myc levels even in the presence of cycloheximide, indicating that these compounds accelerated c-Myc degradation (Fig. 2C). To analyze the degradation pathway after E-H1 cells treated with DOX were treated with the compounds and/or the proteasome inhibitor MG-132, c-Myc was immunoprecipitated, and its ubiquitination was examined by immunoblotting with an anti-ubiquitin antibody. We found that c-Myc ubiquitination was accelerated by the addition of these compounds (Fig. 2D). Therefore, we concluded that these compounds inhibit c-Myc transcriptional activity by accelerating the ubiquitin-d ependent protein degradation of c-Myc, as is the case with antimycin A (manuscript under revision).

### Endogenous c-Myc protein degradation by **1** and **2** in cancer cells

**Figure 3:**
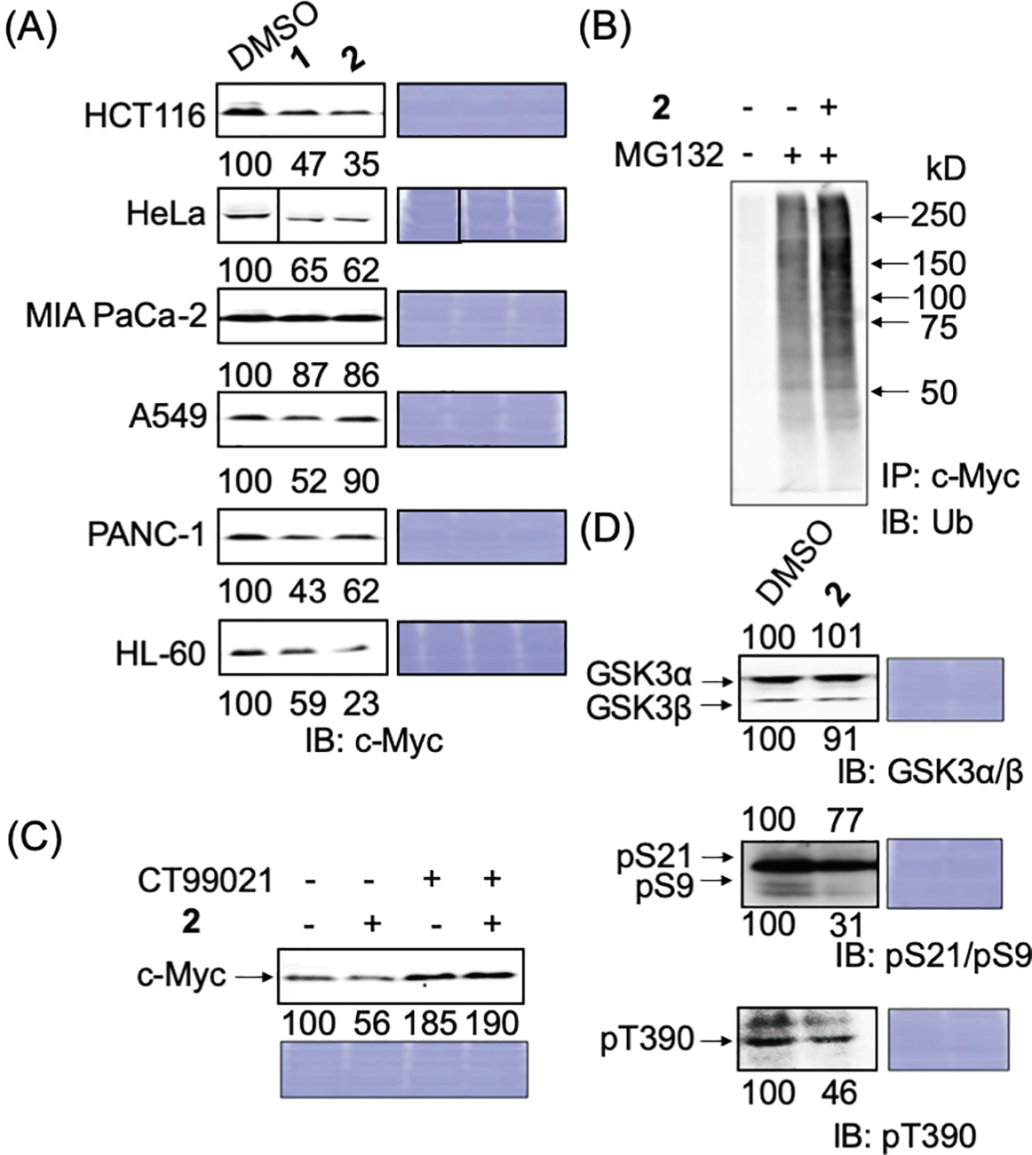
Degradation mechanism of endogenous c-Myc protein by compounds **1** and **2** in cancer cells. (A) c-Myc levels of c-Myc protein after hit compounds **1** and **2** treatment in different cancer cells. Cells were harvested and lysed after treatment with compounds **1** or **2** (50 ng/mL) for 24 h and analyzed by immunoblotting. Coomassie brilliant blue (CBB) staining was used as the loading control. Quantification of c-Myc levels was performed using ImageJ software and is shown as the percentage of cells treated with DMSO alone. (B) Increased ubiquitination of c-Myc by compound **2** in HCT116 cells. Cells were harvested and lysed after treatment with compound **2** (50 ng/mL), with or without MG-132 (6 μM), for 6 h. c-Myc proteins in each sample were harvested from 300 μg lysates using anti-c-Myc antibody-conjugated beads, and their ubiquitination was analyzed by immunoblotting. Representative results from two independent experiments are shown. (C) c-Myc protein reduction by compound **2** was recovered by CT99021 (10 μM) in HCT116 cells. HCT116 cells were treated with or without CT99021 and/or compound **2** (50 ng/mL) for 24 h, harvested, and lysed, and c-Myc levels were analyzed by immunoblotting. CBB staining was used as the loading control. Protein levels were quantified using ImageJ and are shown as the percentage of cells without antimycin A or the compounds. Representative results from two independent experiments are shown. (D) Effect of compound **2** on the levels of GSK3α/β and their phosphorylation in HCT116 cells. Cells were harvested and lysed after treatment with compound **2** (50 ng/mL) for 24 h. GSK3α/β levels and their phosphorylation levels (pS21/9 of GSK3α/β and pT390 of GSK3β) were analyzed by immunoblotting. CBB staining was used as the loading control. Protein levels were quantified using ImageJ and are shown as a percentage of cells treated with DMSO alone. Representative results from two independent experiments are shown.

We also examined whether these compounds accelerated the degradation of endogenous c-Myc in cancer cells. When HCT116 (colorectal cancer), HeLa (cervical cancer), MIA PaCa-2 (pancreatic cancer), A549 (lung cancer), PANC-1 (pancreatic cancer), and HL-60 (leukemia) cells were treated with these compounds for 24 h, and the endogenous c-Myc levels were examined, we found that the c-Myc levels decreased in all cancer cells; however, the extent of this decrease varied among the cells (Fig. 3A).

The factor(s) that is/are responsible for the sensitivity is currently being examined but is yet to be elucidated. Among c-Myc proteins, degradation was most apparent in HCT116 cells; we selected these for further examination.

### Ubiquitin-dependent degradation of c-Myc by compound **2** through GSK3 activation

After purification, because the amount of **2** (3.3 mg) was greater than that of **1** (1.4 mg), **2** was selected for further examination.

We examined whether **2** degrades c-Myc in a ubiquitin–proteasome-dependent manner in HCT116 cells, as in E-H1 cells. After HCT116 cells were treated with **2** and/or the proteasome inhibitor MG-132, c-Myc was immunoprecipitated, and its ubiquitination was examined by immunoblotting with an anti-ubiquitin antibody. The ubiquitination of c-Myc increased in the presence of **2**, indicating that **2** accelerated the degradation of c-Myc in a ubiquitin–proteasome-dependent manner in HCT116 cells (Fig. 3B).

Next, we examined whether the GSK3α/β dependent pathway caused this c-Myc reduction since GSK3 is the most responsible protein kinase for the ubiquitin-dependent degradation of c-Myc. Congruently, we have already found that antimycin A induced the degradation of c-Myc through the activation of GSK3 (manuscript under revision). When a GSK3 inhibitor, CT99021, was added together with **2** to HCT116 cells, the reduction in c-Myc caused by **2** was stopped, indicating that **2** degrades c-Myc dependently in a GSK3-dependent manner (Fig. 3C). Then, the phosphorylation status of GSK3α/β was examined. We found that the inhibitory phosphorylations at S21 of GSK3α, S9 of GSK3β, and T390 of GSK3β were reduced (Fig. 3D). These results indicate that compound **2** induced the ubiquitin-dependent degradation of c-Myc through the activation of GSK3α/β.

### Mitochondrial dysfunction induced by **2**

The structure of **2** (or SS49) was similar to that of cytovaricin and oligomycin A (Fig. 1). Cytovaricin and oligomycin A are inhibitors of H^+^-ATP synthase in mitochondrial complex V and abrogate mitochondrial respiratory function. Recently, we identified a natural product with a similar structure, YO-001A, which also inhibits H^+^-ATP synthase in mitochondrial complex V [33]. The effect of **2** on mitochondrial activity was examined using a Seahorse XFe96 analyzer [34].

**Figure 4:**
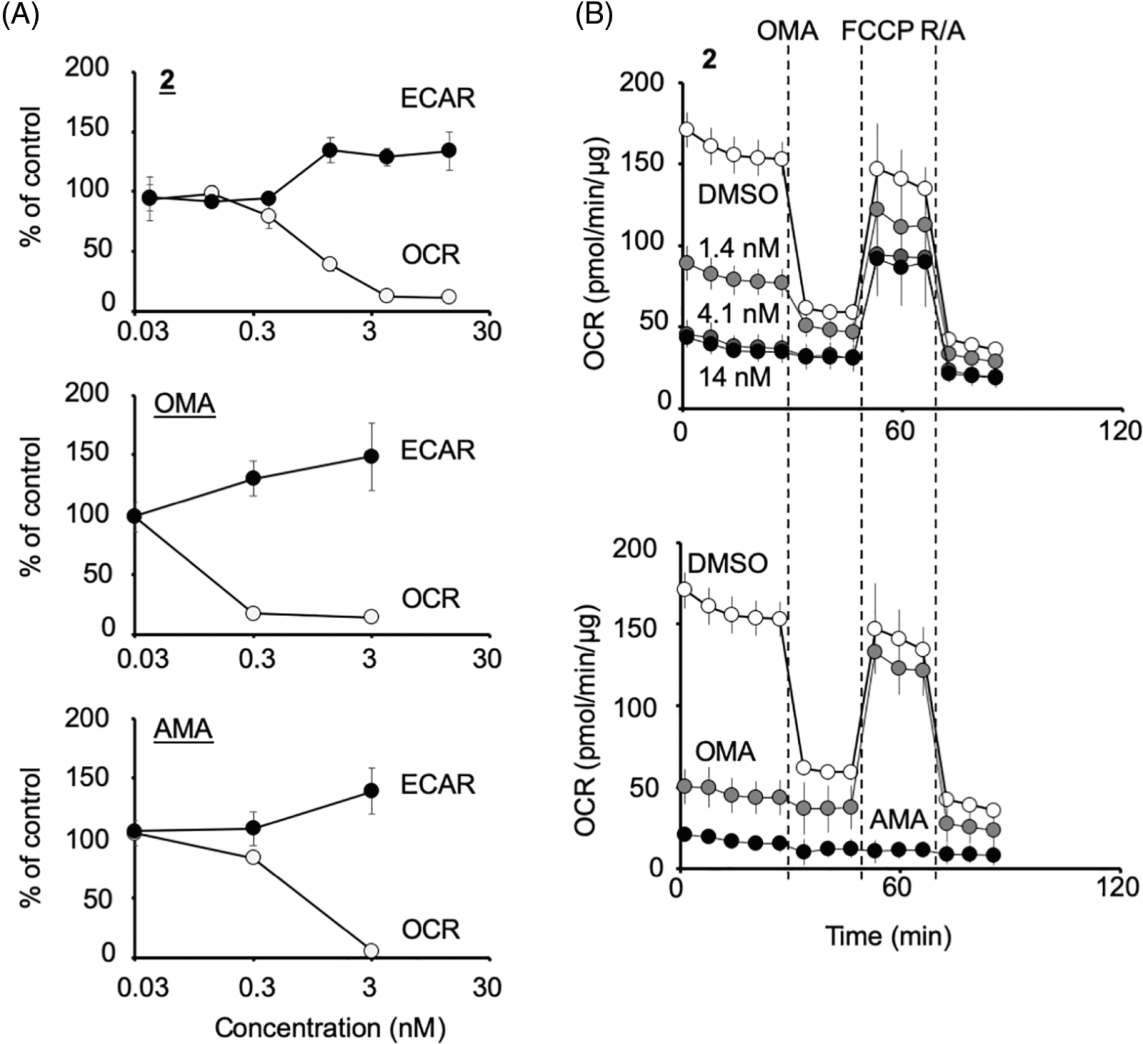
Compound **2** abrogates the mitochondrial function of HCT116 cells by inhibiting the mitochondrial complex V. (A) Percentage changes in oxygen consumption rate (OCR) and extracellular acidification rate (ECAR) (relative to their respective baseline values) induced by compound **2**, oligomycin A (OMA), and antimycin A (AMA) at the concentrations indicated. Each data point represents the mean ± SD (n = 3) at 1.5 h after treatment. A representative experiment from two independent experiments is shown. (B) After the measurement of OCR and ECAR as in (A), the Mito Stress Test was performed, in which OCR values were measured after the addition of oligomycin A (OMA; 1 μM), carbonyl cyanide 4-(trifluoromethoxy)-phenylhydrazone (FCCP; 0.125 μM) and rotenone/antimycin A (R/A; 1 μM each) in a stepwise manner. Data on compound **2** at 1.4, 4.1, or 14 nM treated cells (upper panel) and that of OMA (3 nM)- or AMA (3 nM)-treated cells (lower panel) together with their DMSO control are shown. Each data point represents the mean ± SD (n = 3). A representative experiment from two independent experiments is shown.

HCT116 cells were treated with **2** or the control compounds (antimycin A and oligomycin A) at several concentrations, and the OCR and ECAR were monitored (Fig. 4A). Compound **2** at more than 1 ng/mL (=1.4 nM) significantly reduced the OCR and increased ECAR as oligomycin A or antimycin A at more than 0.3 nM, indicating that **2** abrogates mitochondrial function. In the following Mito Stress Test, **2** treated samples were still sensitive to FCCP, similar to 3 nM oligomycin A but not 3 nM antimycin A, indicating that **2** inhibits mitochondrial complex V (Fig. 4B). The IC_50_ of **2** to inhibit mitochondria was determined to be 0.72 ng/mL (=0.97 nM), which was about eight times weaker than that of oligomycin A (0.12 nM) (Fig. 4A).

### Compound **2** induces the acceleration of c-Myc degradation by GSK3 activation through ROS production via mitochondrial dysfunction

**Figure 5:**
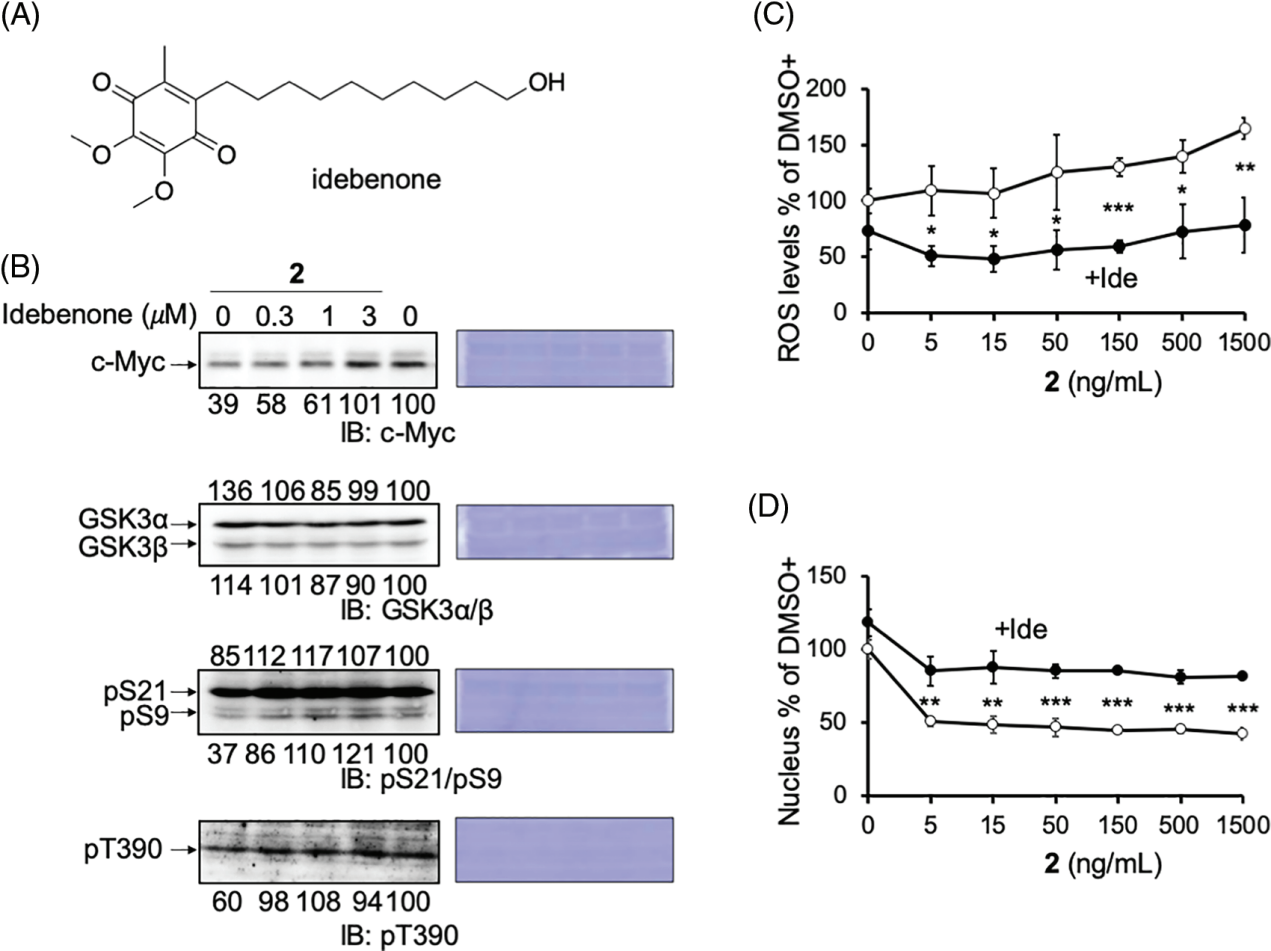
Reactive oxygen species (ROS) produced by compound **2** inhibits cell growth. (A) The structure of idebenone. (B) Reduced c-Myc protein levels and pS9/pT390 of GSK3α/β levels by compound **2** were canceled by idebenone in HCT116 cells. Idebenone was added at the indicated concentrations to compound **2** (50 ng/mL) for 24 h, and the cells were harvested and lysed. The c-Myc protein levels, GSK3α/β levels, and their phosphorylation (pS21/9 and pT390) were analyzed by immunoblotting, quantitated by ImageJ, and shown as the percentage of that of control (DMSO) cells. Coomassie brilliant blue (CBB) staining was used as the loading control. (C) HCT116 cells were treated with compound **2** at the indicated concentration with (closed circle; +Ide) or without (open circle) idebenone (3 μM), cultured for 1 h, fixed, and the mitochondrial ROS levels were measured using the INCA. Results are shown as the mean ± SD (n = 3), and a representative result of two independent experiments is shown. (D) HCT116 cells were treated with compound **2** at the indicated concentrations with (closed circle; +Ide) or without (open circle) idebenone (3 μM), cultured for 24 h, fixed, and cell growth was measured by counting the number of nuclei after Hoechst staining. Results are shown as the mean ± SD (n = 3), and a representative result of two independent experiments is shown. ANOVA test, *p < 0.05, **p < 0.01, ***p < 0.001.

Compound **2** abrogates the mitochondrial function, which may induce ROS production and the activation of GSK3. The effect of ROS reduction by the antioxidant idebenone was examined to evaluate this possibility (Fig. 5A). Idebenone has been widely portrayed as a potent antioxidant that can effectively protect against ROS [35,36]. As expected, when idebenone was added with **2** to HCT116 cells, the reduction in c-Myc protein levels by **2** was recovered dose-dependent (Fig. 5B; c-Myc blot). In addition, the decrease in the inhibitory phosphorylation of GSK3 was recovered by idebenone (Fig. 5B; pS9/pS21 and pT390 blots). These results indicated that the increased ROS production induced by damaged mitochondria activated GSK3α/β and reduced c-Myc protein levels. We also confirmed ROS production by **2** in cell culture. In the presence of **2**, mitochondrial ROS production increased dose-dependent in HCT116 cells, which was canceled in idebenone (Fig. 5C).

Previously, we found that the mitochondrial inhibitor, antimycin A inhibited cancer cell growth in a c-Myc- and ROS-dependent manner (manuscript under revision). Compound **2**, another mitochondrial inhibitor, inhibited the growth of HCT116 cells in a dose-dependent manner. In addition, we found that this inhibition was canceled by idebenone, albeit partially (Fig. 5D). Moreover, we confirmed that oligomycin A could also reduce the c-Myc level in HCT116 cells (Suppl. Fig. S3). These results provide another example that a mitochondrial inhibitor induces ROS-dependent growth inhibition of cancer cells and suggests the involvement of c-Myc in this pathway.

## Discussion

**Figure 6:**
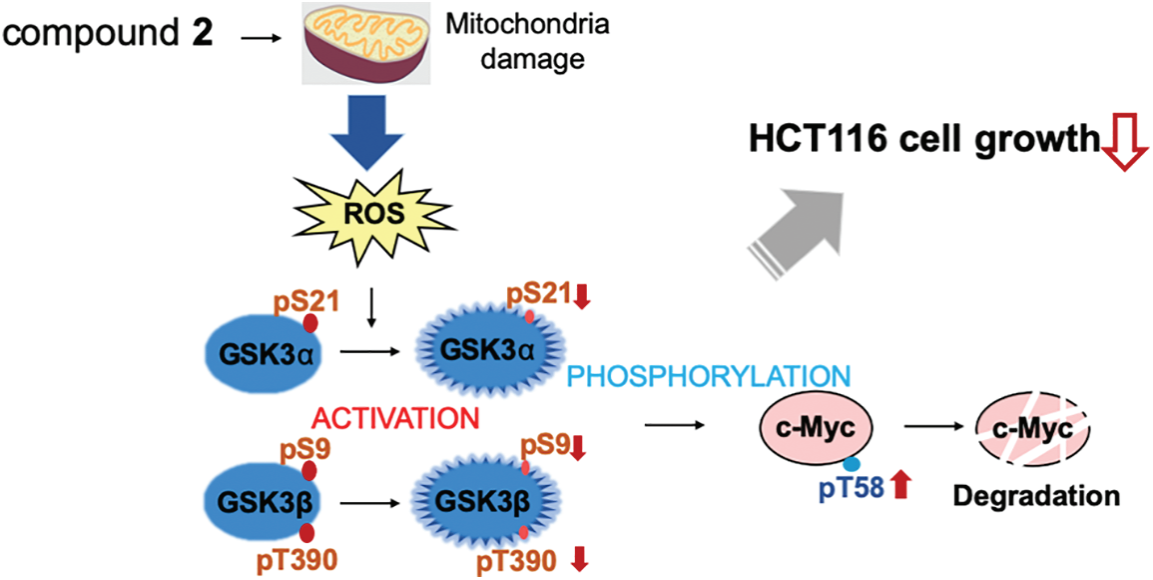
Compound **2** promotes c-Myc protein degradation and inhibits cell growth. Compound **2** caused mitochondrial damage, which inhibited cell growth in HCT116 cells. The damaged mitochondria produce reactive oxygen species (ROS) to activate GSK3α through decreased phosphorylation at S21, and GSK3β through decreased phosphorylation at S9 and T390, which lead to increased phosphorylation at T58 of c-Myc to induce degradation.

c-Myc is a critical regulator of cell proliferation and growth. Herein, we purified and identified the active compounds from a broth extract produced by a strain of *Streptomyces* sp., RK19-A0402, a c-Myc inhibitor identified using a high-throughput screening system. Compounds **1** and **2** enhanced the ubiquitin-dependent degradation of c-Myc. The planar structure of **2** was identical to that of SS49. Compound **1** appeared to be an epimer of the hydroxyl group at the C-34 position (Suppl. Table S1). SS49 is closely related to oligomycin A, YO-001A, and the aglycone of cytovaricin. However, its biological activity has not yet been studied [31,33,37]. We have shown that **2** inhibits mitochondrial complex V and induces mitochondrial dysfunction like oligomycin A. ROS production by the damaged mitochondria activated GSK3α/β, phosphorylating c-Myc for ubiquitin-dependent degradation, which is responsible for the growth inhibition of cancer cells (Fig. 6). In addition to the isolation of antimycin A, this study provides a theoretical basis for developing an anticancer drug screening system using c-Myc as a target.

HCT116 cells, a colorectal cancer cell line enriched with c-Myc protein, showed strong sensitivity to **2** treatments. In HCT116 cells, **2** inhibited c-Myc by protein degradation through the ROS-mediated activation of GSK3α/β. This is consistent with our previous finding that c-Myc-enriched cancer cells are sensitive to another mitochondrial inhibitor, antimycin A (manuscript under revision). Our results provide another implication for the pathophysiology of cancer. In this context, we did not detect any increase in the phosphorylation of S62, which is known to be mainly phosphorylated by Erk; however, it is also possible that the activation of the Ras-Erk pathway may also contribute to the c-Myc degradation by compound **2** (data not shown). This contribution of the activation of the Ras-Erk pathway by compound **2** needs to be elucidated in the future. In addition, it is known that the activated GSK3 induces phosphorylation and proteasomal degradation of β-catenin, and c-Myc is one of the major target genes of β-catenin. It is also possible that compound **2** may affect the c-Myc translation through the mRNA reduction through the β-catenin decrease, specifically in cancer cells. However, this possibility is excluded in E-H1 cells. The effect of compound **2** on the c-Myc translation in cancer cells also needs to be elucidated.

Oxidative stress has been implicated in cancer pathophysiology [38–40]. Increased ROS levels can induce apoptosis in cancer cells, underlining the potential role of ROS modulation in anticancer combinatorial therapies. ROS induction is one approach to cancer therapy [40–42]. We examined cell growth and ROS production induced by damaged mitochondria. These results suggested that **2** induced ROS production and inhibited cell growth through mitochondrial damage. It is reported that other ROS-producing compounds also activate GSK3α/β. Dexamethasone enhanced the ROS levels in osteoblasts and promoted the activation of GSK3β in osteoblasts [43]. A stress response protein, regulated in DNA damage and development 1 (REDD1), promotes ROS, and the addition of H_2_O_2_ activates GSK3α/β through the suppression of the pS21/9 of GSK3α/β [44,45]. Our finding of ROS production by **2** may add another example of the activation pathway of GSK3α/β responsible for the c-Myc degradation and growth inhibition of cancer cells.

## Supplementary Materials

Table S1 ^1^H and ^13^C NMR chemical shifts in CDCl_3_

Figure S1 The c-Myc transcriptional inhibition by hit compounds 1 and 2 in E-H1 cells.

Figure S2 Effects of hit compounds 1 and 2 on HNF1B levels in D-D1 cells.

Figure S3 Effects of oligomycin A on c-Myc levels in E-H1 cells.

## Data Availability

All data supporting the conclusions of this study have been included in this article. Please contact the authors for requests for raw data.
