# Supplementary material for: Identification of microbial metabolites that accelerate the ubiquitin-dependent degradation of c-Myc
Source: Oncol Res. 2023 Jul 21;31(5):655–66. doi: 10.32604/or.2023.030248 (PMC10398403; doi:10.32604/or.2023.030248)
Supplement: Table S1 [file OncolRes-31-30248-s001.pdf]

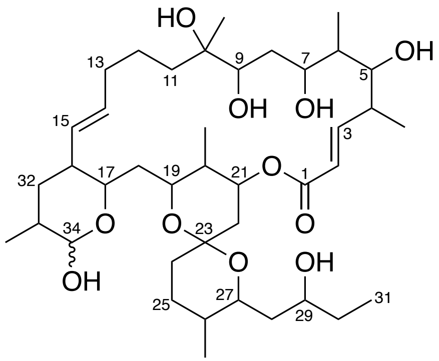

Table S1. ^{1}H and ^{13}C NMR chemical shifts in $CDCl_3$

Position	2			1	Position	2			1
	δ_C	δ_H	mult (*J* in Hz)	δ_C		δ_C	δ_H	mult (*J* in Hz)	δ_C
1	165.3	-		ND	21	70.6	5.25		70.8
2	122.3	5.76	d (15.5)	ND	22	35.6	1.64		ND
3	151.6	6.65	dd (15.5, 10.3)	ND			1.80	dd (13.2, 5.2)	
4	41.3	2.41		41.3	23	97.4	-		ND
5	81.2	3.65	brd (9.8)	81.2	24	30.1	1.49		ND
6	36.8	1.42		ND			1.65		
7	77.9	4.07	brd (6.9)	78.0	25	26.9	1.42		26.9
8	35.4	1.28		35.4			2.11		
		1.58			26	31.1	1.57		31.3
9	78.3	3.48		78.6	27	68.7	3.86		66.7
10	75.4	-		ND	28	41.4	1.21		ND
11	37.6	1.20		ND			1.59		
		1.36			29	69.7	3.86		69.6
12	22.6	1.48		22.6	30	30.8	1.46	2H	30.9
		1.63			31	10.1	0.94	3H, t (6.9)	10.2
13	33.0	1.92		32.5	32	38.7	1.08		ND
		2.38					1.67		
14	132.3	5.09	ddd (14.9, 9.8, 1.7)	ND	33	37.0	1.50		ND
15	132.7	5.26		ND	34	101.5	4.28	d (8.6)	94.8
16	47.2	1.88		47.9	Me-4	17.6	1.16	3H, d (8.9)	17.7
17	74.1	3.45		ND	Me-6	4.0	0.85	3H, d (7.5)	4.0
18	36.7	1.29		ND	Me-10	20.2	1.09	3H, s	20.2
		1.90			Me-20	6.3	0.62	3H, d (6.9)	6.2
19	64.1	3.87		64.2	Me-26	11.7	0.91	3H, d (6.9)	11.5
20	34.9	1.99		34.7	Me-33	16.8	0.92	3H, d (8.9)	17.0

500 MHz for ^{1}H, 125 MHz for ^{13}C, ND: not determined
