# Supplementary material for: Identification of microbial metabolites that accelerate the ubiquitin-dependent degradation of c-Myc
Source: Oncol Res. 2023 Jul 21;31(5):655–66. doi: 10.32604/or.2023.030248 (PMC10398403; doi:10.32604/or.2023.030248)
Supplement: Figure S1 [file OncolRes-31-30248-s001.docx]

**
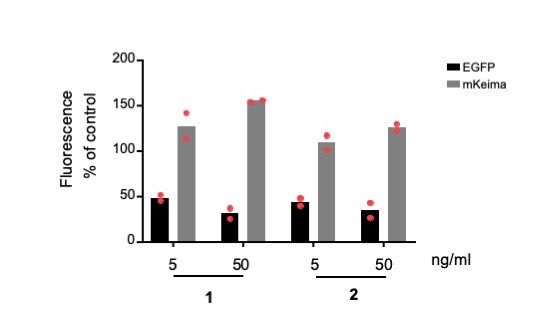
**

**Figure S1. The c-Myc transcriptional inhibition by hit compounds 1 and 2 in E-H1 cells.**

E-H1 cells were fixed 24 h after adding 100 ng/mL doxycycline and **1** or **2**. The mean of relative values [% of control (DOX+)] are shown. Results were obtained from two independent experiments. The red dots indicate individual measurements.


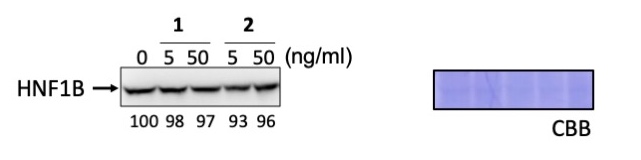


**Figure S2. Effects of hit compounds 1 and 2 on HNF1B levels in D-D1 cells.**

D-D1 cells were harvested and lysed after treatment with 100 ng/mL DOX and compounds **1** or **2** for 24 h, and HNF1B levels were analyzed by immunoblotting. Coomassie brilliant blue (CBB) staining was used as the loading control. Protein levels were quantified using ImageJ software and are shown as the percentage of cells treated with DMSO. Results were obtained from two independent experiments.

**
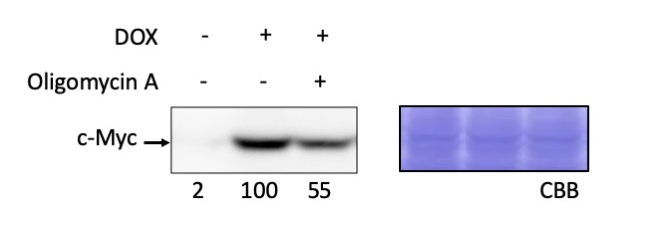
**

**Figure S3. Effects of oligomycin A on c-Myc levels in E-H1 cells.**

E-H1 cells were harvested and lysed after treatment with 100 ng/mL DOX and oligomycin A (5 nM) for 24 h, and c-Myc levels were analyzed by immunoblotting. Coomassie brilliant blue (CBB) staining was used as the loading control. Protein levels were quantified using ImageJ software and are shown as the percentage of cells treated with DMSO. Results were obtained from two independent experiments.
